# Automatic segmentation of fat metaplasia on sacroiliac joint MRI using deep learning

**DOI:** 10.1186/s13244-024-01659-y

**Published:** 2024-03-26

**Authors:** Xin Li, Yi Lin, Zhuoyao Xie, Zixiao Lu, Liwen Song, Qiang Ye, Menghong Wang, Xiao Fang, Yi He, Hao Chen, Yinghua Zhao

**Affiliations:** 1 Department of Radiology, The Third Affiliated Hospital of Southern Medical University (Academy of Orthopedics, Guangdong Province), Guangzhou, 510630 Guangdong China; 2 Department of Computer Science and Engineering, The Hong Kong University of Science and Technology, Hong Kong, 999077 China; 3 Department of Rheumatology and Immunology, The Third Affiliated Hospital of Southern Medical University (Academy of Orthopedics, Guangdong Province), Guangzhou, 510630 China

**Keywords:** Axial spondyloarthritis, Deep learning, Fat metaplasia, Magnetic resonance image, Sacroiliac joint

## Abstract

**Objective:**

To develop a deep learning (DL) model for segmenting fat metaplasia (FM) on sacroiliac joint (SIJ) MRI and further develop a DL model for classifying axial spondyloarthritis (axSpA) and non-axSpA.

**Materials and methods:**

This study retrospectively collected 706 patients with FM who underwent SIJ MRI from center 1 (462 axSpA and 186 non-axSpA) and center 2 (37 axSpA and 21 non-axSpA). Patients from center 1 were divided into the training, validation, and internal test sets (*n* = 455, 64, and 129). Patients from center 2 were used as the external test set. We developed a UNet-based model to segment FM. Based on segmentation results, a classification model was built to distinguish axSpA and non-axSpA. Dice Similarity Coefficients (DSC) and area under the curve (AUC) were used for model evaluation. Radiologists’ performance without and with model assistance was compared to assess the clinical utility of the models.

**Results:**

Our segmentation model achieved satisfactory DSC of 81.86% ± 1.55% and 85.44% ± 6.09% on the internal cross-validation and external test sets. The classification model yielded AUCs of 0.876 (95% CI: 0.811–0.942) and 0.799 (95% CI: 0.696–0.902) on the internal and external test sets, respectively. With model assistance, segmentation performance was improved for the radiological resident (DSC, 75.70% vs. 82.87%, *p* < 0.05) and expert radiologist (DSC, 85.03% vs. 85.74%, *p* > 0.05).

**Conclusions:**

DL is a novel method for automatic and accurate segmentation of FM on SIJ MRI and can effectively increase radiologist’s performance, which might assist in improving diagnosis and progression of axSpA.

**Critical relevance statement:**

DL models allowed automatic and accurate segmentation of FM on sacroiliac joint MRI, which might facilitate quantitative analysis of FM and have the potential to improve diagnosis and prognosis of axSpA.

**Key points:**

• Deep learning was used for automatic segmentation of fat metaplasia on MRI.

• UNet-based models achieved automatic and accurate segmentation of fat metaplasia.

• Automatic segmentation facilitates quantitative analysis of fat metaplasia to improve diagnosis and prognosis of axial spondyloarthritis.

**Graphical Abstract:**

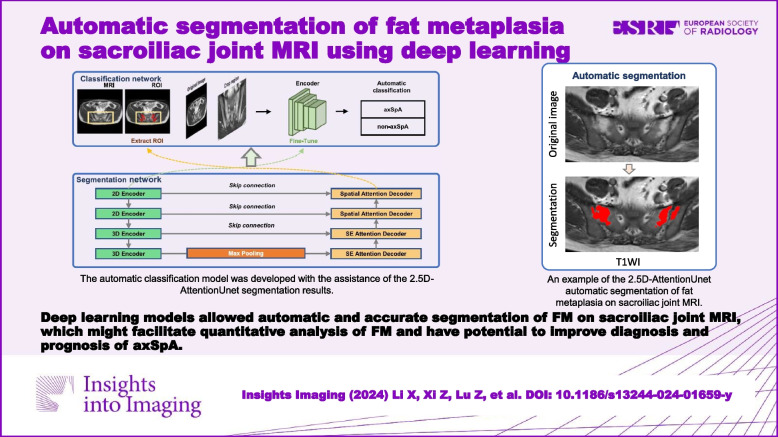

**Supplementary Information:**

The online version contains supplementary material available at 10.1186/s13244-024-01659-y.

## Introduction

Axial spondyloarthritis (axSpA) is a chronic inflammatory disease that typically begins in the sacroiliac joints (SIJs) [1]. Magnetic resonance image (MRI) is the standard imaging modality for early detection of SIJ changes of axSpA, including inflammation (represented by bone marrow edema) and structural damage (such as erosion, fat metaplasia (FM), sclerosis, and ankylosis) [2, 3]. Besides inflammation, contextual information of structural damage enhances axSpA diagnosis [4]. FM is the second most common structural damage, occurring in more than a quarter of axSpA and showing the highest specificity for axSpA diagnosis [3, 5]. Furthermore, ankylosis progression of SIJs and the spine usually follows the onset of FM in SIJs, resulting in loss of function and poorer quality of life [6, 7]. Therefore, analysis of FM in SIJ MRI is crucial for the diagnosis and prognosis of axSpA.

For FM analysis, T1-weighted imaging is a routinely used imaging sequence [3]. On SIJ T1W images, FM indicative of axSpA typically appears as a bright and well-defined lesion in the subchondral bone [3]. Semi-quantitative scoring methods for FM based on visual assessment have been developed for prognosis evaluation of axSpA [8]. However, FM can also occur in 13.8% of non-axSpA [9], such as undifferentiated arthritis, psoriatic arthritis, gouty arthritis, and so on [10]. Furthermore, with increasing age, the distribution patterns of FM commonly change from focal to extensive [11]. These complex situations might mislead the radiologist’s visual interpretation of FM indicative of axSpA and cause incorrect diagnosis or prognostic evaluation. Recently, quantitative analysis of segmented FM using proton density fat fraction based on chemical shift-encoded MRI has been proposed to provide deeper insights into the association between FM and axSpA diagnosis and prognosis [12, 13]. However, the widespread clinical application of quantitative analysis of FM urgently requires robust and accurate automatic segmentation methods.

Deep learning (DL) utilizes multiple layers to automatically learn features from raw images and is widely used in automatic segmentation, classification, and detection for medical image analysis [14]. Several studies reported the success of MRI-based DL algorithms for detecting inflammation and structural damage in SIJs of axSpA [15–17]. Recent studies demonstrated that DL models using the popular UNet architecture had high performance for automatic segmentation of orbital, subcutaneous, and visceral fat [18, 19]. UNet is one of the most popular convolutional neural networks (CNNs) that is robust and efficient in image segmentation [20]. However, the feasibility of using DL methods for automatic segmentation of FM on SIJ MRI has not been fully explored.

This study aimed to develop DL models for automatic segmentation of FM on SIJ MRI and further developed DL models to classify axSpA from non-axSpA based on FM. We also evaluated the clinical utility of the models in assisting radiologists.

## Materials and methods

The institutional review board approved this retrospective study of center 1 (IRB number: 201501003) followed by center 2. Written informed consent was waived for all patients because of the retrospective nature of the study. The study was conducted according to the Checklist for Artificial Intelligence in Medical Imaging (CLAIM) guideline (Supplementary Materials II ) [21].

### Study participants

**Fig. 1:**
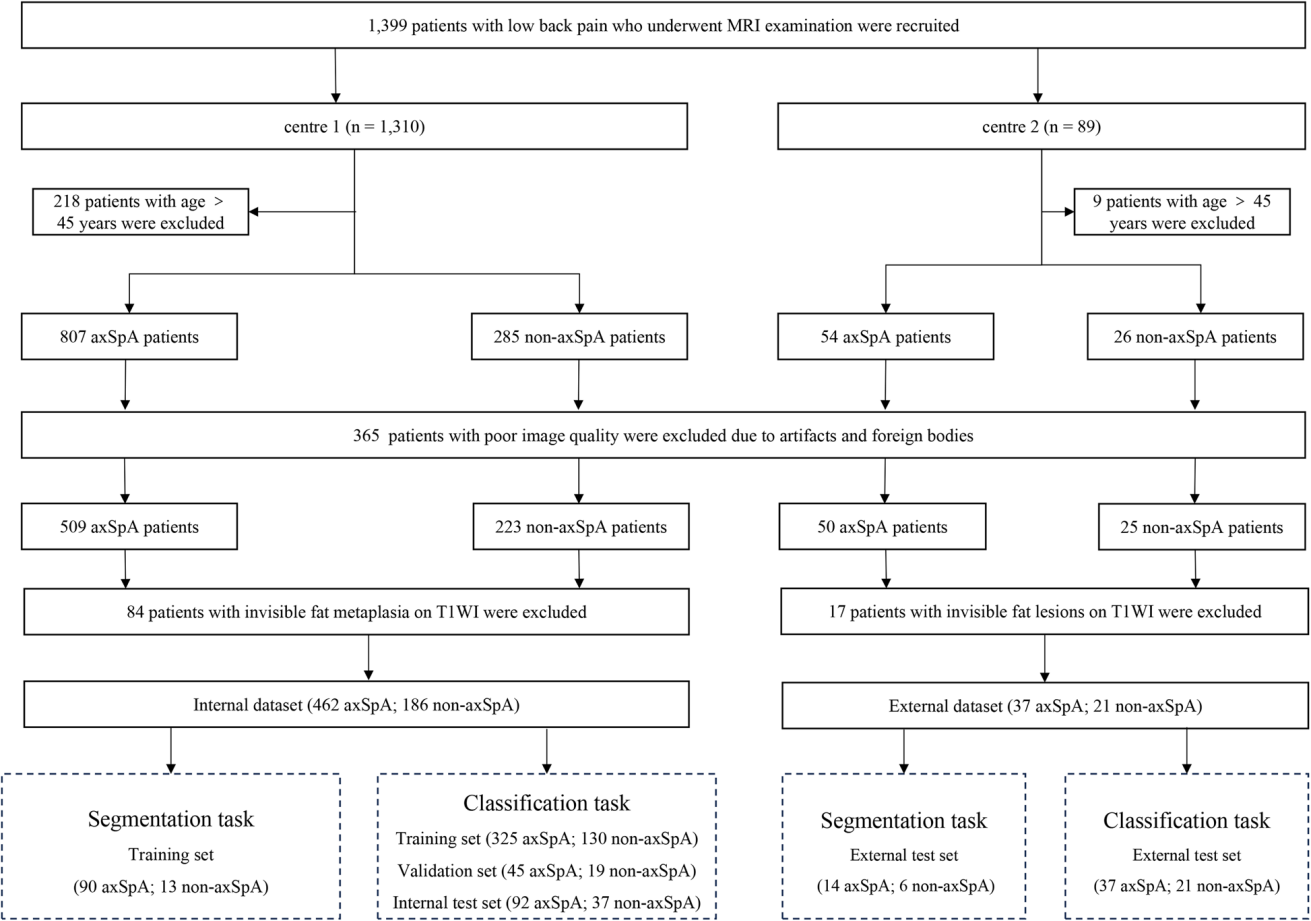
Flowchart of patient inclusion and exclusion. *T1WI* T1-weighted images; *axSpA* axial spondyloarthritis; *non-axSpA* non-axial spondyloarthritis

We enrolled patients from March 2011 to January 2022 in center 1 and from July 2011 to August 2021 in center 2, respectively. The inclusion criteria were patients (a) who had chronic low back pain; (b) with axSpA or non-axSpA diagnosed by rheumatologists; and (c) with SIJ axial T1-weighted images (T1WI), T2-weighted images (T2WI), fat-saturated (FS) T2WI, and available clinical data. FS T2WI techniques included T2 STIR, T2 SPAIR, and T2 DIXON techniques. Details of non-axSpA were described in Supplementary Materials I: Appendix Table 1. The exclusion criteria were as follows: (a) patients with age > 45 years; (b) patients with no FM on MRI observed by radiologists; and (c) MR image quality was poor due to artifacts and foreign bodies. Image quality was considered poor if radiologists (Q.Y. and Y.H.Z. with 12 and 31 years of radiology reading experience, respectively) consensually agreed that segmentation of the radiograph could not be performed for meaningful analysis. The flowchart of patient inclusion and exclusion is shown in Fig. 1. After patient exclusion, we finally included 648 patients (462 axSpA and 186 non-axSpA) from center 1 and 58 patients (37 axSpA and 21 non-axSpA) from center 2, respectively. For the segmentation task, 103 patients were randomly selected from center 1 as the training set with a fourfold cross-validation strategy; 20 patients randomly selected from center 2 were used as the external test set. For the classification task, we divided patients from center 1 into the training (*n* = 455), validation (*n* = 64), and internal test sets (*n* = 129); and used all 58 patients from center 2 as the external test set. All clinical characteristics, including age, gender, human leukocyte antigen-B27 (HLA-B27) status, erythrocyte sedimentation rate (ESR), and C-reactive protein (CRP) concentration, were collected from the electronic health records.

### Image analysis

**Fig. 2:**
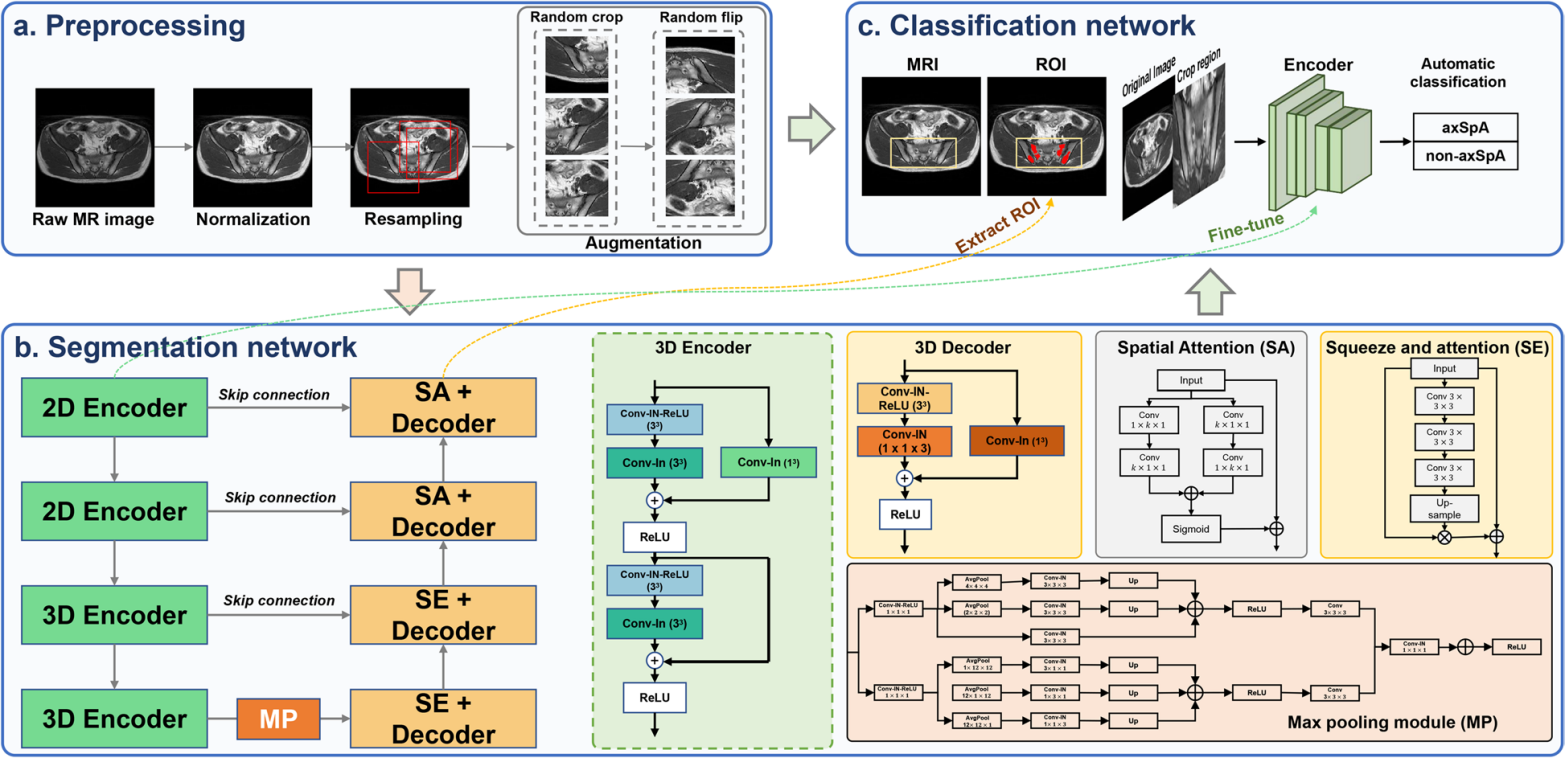
Framework of the deep-learning-based segmentation and classification models. The 2.5D-AttentionUnet segmentation model was developed by combining two- and three-dimensional convolution. The automatic classification model was developed with the assistance of the segmentation results.* axSpA* axial spondyloarthritis; *non-axSpA* non-axial spondyloarthritis; *ROI* region of interest

The MRI protocols are shown in Supplementary Materials I: Appendix Table 2. The 2009 Assessment of SpondyloArthritis International Society (ASAS) considers that both axial and coronal oblique views of SIJ MRI are available [22]. All patients who underwent sacroiliac joint MRI in two centers had T1 oblique axial, T2 oblique coronal, and T2 fat-suppressed oblique coronal images. Axial oblique T1WI, coronal oblique T2WI, and coronal oblique FS T2WI were downloaded and stored as Digital Imaging and Communications in Medicine (DICOM) files. Patient-protected health information was deleted from DICOM data to meet the US (HIPAA), European (GDPR), or Other Relevant Legal Requirements [23]. According to evaluation criteria defined by the ASAS MRI working group (Supplementary Materials I: Appendix method 1) [3], the region of interest (ROI) of FM was manually delineated for each slice on the SIJ T1WI of all patients using ITK-SNAP software (version 3.6.0; www.itk-snap.org) by two radiologists (Y.Y.S. and X.L.), who have 3 and 5 years of musculoskeletal experience. Then, the ROIs were reviewed and corrected in concordance between the two senior radiologists (Q.Y. and Y.H.Z.). MR images on all data sets were preprocessed before inputting into the model (Fig. 2a and Supplementary Materials I: Appendix method 2). The initially determined ROIs by two senior radiologists are used as inputs to the trained segmentation model. The output segmentation masks of the trained model were corrected again by all radiologists for the calibration of ROIs. This human-model interaction was repeated five times, and the final determined ROIs were used as the ground truth for the 2.5D-AttentionUnet model’s segmentation task. Two senior radiologists (Q.Y. and Y.H.Z.) independently reviewed the electronic health records and annotated participants as axSpA or non-axSpA patients.

### Model development

We developed a novel 2.5D-AttentionUnet model specifically designed for the segmentation of FM on SIJ MRI. This model effectively combines the capabilities of 2D and 3D CNNs to extract hierarchical image features. Additionally, we employ the attention mechanism to accurately capture metaplasia regions of various shapes and sizes. We utilized spatial attention [24] and squeeze-and-attention [25] to learn the relative importance of each spatial position in the encoded features (Supplementary Materials I: Appendix method 3). The spatial attention mechanism captures the spatial distribution of encoded features, highlighting important features and suppressing less significant ones. The squeeze-and-attention mechanism involves reducing feature dimensionality through global average pooling (squeeze operation) and obtaining attention weights for each channel using an activation function (excitation operation). These attention weights are then applied to the encoded features, resulting in the final feature representation. The cross-entropy loss, Dice score loss, and attention loss [26] were adopted as the loss functions for 2.5D-AttentionUnet. The Adam algorithm was used as the optimizer with a batch size of 2 during training. The model was trained for 400 epochs, with the learning rate initialized to 5e − 4 and halved every 3800 iterations. We have also trained the 2D-UNet, 3D-UNet, ResUNet, UNETR [27], and Attention UNet [28] models to compare their segmentation performance with our 2.5D-AttentionUNet model (Fig. 2b and Supplementary Materials I: Appendix method 3).

Furthermore, we developed a classification model to distinguish axSpA from non-axSpA based on the best segmentation model. Specifically, the ROI of FM on the segmentation mask was cropped to generate an ROI map for the original image. The ROI map was then concatenated with the original image to be fed into the classification model. The binary cross-entropy loss was used for the classification model. We trained the model for 300 epochs with an initial learning rate of 0.001. The Gradient-weighted Class Activation Mapping (Grad-CAM) method was used to visualize essential response areas during the classification. Further details of the classification development of the DL model are presented in Fig. 2c and Supplementary Materials I: Appendix method 4.

Two Intel Xeon gold 5220R 2.2 GHz central processing units with 16 × 64 GB of double-data-rate-4 synchronous dynamic random-access memory and a GeForce RTX 3090 Ti graphics processing unit (Nvidia, Santa Clara, CA, USA) were used and ran on a Linux system (Ubuntu, version 18.04) with a CUDA version 11.1 platform. Our DL models were implemented using MONAI open-source libraries (version 0.9.0) and the PyTorch package (version 1.10.0), based on open-source software (Python, version 3.8.0; Python Software Foundation, Wilmington, DE, USA). The training parameters and source code can be found online (https://github.com/hust-linyi/2.5D-AttentionUNet).

### Radiologist evaluation

The T1W images on the external test set were evaluated by a radiological resident (M.H.W. with 3 years of musculoskeletal experience) and an expert radiologist (R.Z. with 17 years of musculoskeletal experience) who were blinded to the ground truth. They independently segmented FM lesions and classified the cases as axSpA or non-axSpA at baseline. After 3 months, they re-evaluated the images with the assistance of DL models.

### Statistical analysis

Statistical analyses were performed using the SPSS (version 23.0, IBM, Armonk, NY, USA) and Python (version 3.8.0). Continuous variables were evaluated with the Mann–Whitney *U* test, and categorical data were assessed with the chi-square test. The performance of the segmentation models was evaluated using the Dice similarity coefficient (DSC), precision, and recall. The performance of the classification model was measured using the area under the receiver operating characteristic curve (AUC), accuracy, sensitivity, specificity, positive predictive value (PPV), negative predictive value (NPV), and Cohen’s *κ* value. The DSC and accuracy were compared between radiologists without and with model assistance using the Wilcoxon signed rank test or the chi-square test, as appropriate. The bootstrap technique was used to calculate 95% confidence intervals (CI). Two-sided *p* < 0.05 was considered significant.

## Results

### Patient characteristics

A total of 706 patients (median age 28 years, interquartile range 23–34 years; 519 (73.5%) males) were finally included for analysis. For the segmentation task, there was a significant difference with a *p* value of < 0.05 in CRP concentration among the total data set, training, and external test sets, but others were not (Table 1). For the classification task, all clinical characteristics except for ESR and CRP concentration were not statistically different between patients among the total data set, training, validation, internal test, and external test sets (all *p* > 0.05) (Supplementary Materials I: Appendix Table 3).

**Table 1:** Patient characteristics for the segmentation task

Clinical characteristics	All (*n* = 123)	Training set (*n* = 103)	Test set (*n* = 20)	*p* value
Age (years)	26.0 (23.0, 32.0)	27.0 (24.0, 32.0)	23.0 (19.3, 30.8)	0.190
Disease duration (months)	24.00 (7.0, 72.0)	24.00 (8.0, 72.0)	13 (1.0, 75.0)	0.038
Sex				0.797
Male	89 (72.36)	75 (72.82)	14 (70.0)	
Female	34 (27.64)	28 (27.18)	6 (30.0)	
HLA-B27				0.653
( +)	80 (65.0)	70 (67.9)	10 (50.0)	
( −)	25 (20.3)	21 (20.4)	4 (20.0)	
Missing	18 (14.7)	12 (11.7)	6 (30.0)	
ESR (mm/H)	20.0 (10.0, 40.0)	20.0 (9.0, 35.0)	43.5 (17.3, 62.0)	0.027
CRP (mg/L)	7.0 (2.7, 22.1)	6.9 (2.2, 19.1)	24.7 (4.9, 40.7)	0.158

Categorical variables are presented as numbers with percentages in parentheses, and continuous variables are shown as medians with interquartile in parentheses

*HLA-B27* human leukocyte antigen-B27, *ESR* erythrocyte sedimentation rate, *CRP* C-reactive protein, *mm/H* millimeter per hour, *mg/L* milligram per liter, *n* number

### Performance of the 2.5D-AttentionUNet segmentation model

The 2D-UNet, 3D-UNet, ResUNet, UNETR, and AttentionU-Net models achieved DSCs of 76.42–80.05% for segmentation of FM on internal cross-validation and 57.81–67.40% on the external test set. Compared with those segmentation models, our novel 2.5D-AttentionUNet model showed the best performance with DSCs of 81.86% ± 1.55% and 85.44% ± 6.09% on internal cross-validation and the external test set (all *p* < 0.05, except for 3D-UNet model) (Table 2). On the external test set, there were only 2 cases where the DSC obtained by the model was below 80.00%, one of which was axSpA (DSC, 63.24%) and the other was non-axSpA (DSC, 79.65%).

**Table 2:** Performance of deep learning segmentation models

Models	Internal cross-validation			External test set		
	DSC (%)	Precision (%)	Recall (%)	DSC (%)	Precision (%)	Recall (%)
2.5D-AttentionUNet (ours)	81.86 ± 1.55	80.49 (80.16–80.82)	85.5 (85.34–85.66)	85.44 ± 6.09	85.83 (82.62–89.04)	86.43 (81.10–91.76)
2D-UNet	76.97 ± 2.11**	78.5 (78.25–78.75)	78.24 (77.84–78.64)	66.53 ± 19.37**	69.14 (62.20–74.04)	68.09 (59.71–76.50)
3D-UNet	80.05 ± 1.57	80.18 (79.71–81.55)	82.4 (82.00–82.80)	67.40 ± 20.84**	85.12 (80.31–89.37)	62.87 (52.46–71.26)
ResUNet	76.42 ± 1.92 **	76.49 (76.15–76.83)	79.56 (79.31–79.81)	66.41 ± 17.60**	75.74 (69.66–79.58)	65.66 (57.32–72.96)
UNETR	73.94 ± 2.68 **	79.64 (79.12–80.16)	72.93 (72.51–73.35)	57.81 ± 21.58**	68.83 (61.45–77.27)	55.26 (46.22–67.42)
Attention U-Net	79.73 ± 1.65 *	79.95 (79.59–80.31)	82.47 (82.12–82.82)	65.54 ± 24.25*	72.29 (63.05–76.22)	67.95 (56.84–76.66)

DSC is presented as an average percentage with a standard deviation. Precision and recall are shown as percentages with 95% confidential intervals. Paired *t*-tests are performed to determine the statistical significance of differences between the 2.5D-AttentionUNet and other models

*DSC* Dice similarity coefficient

^*^represents *p* value < 0.05

^**^represents* p* value < 0.001

### Clinical utility of the 2.5D-AttentionUNet segmentation model

**Fig. 3:**
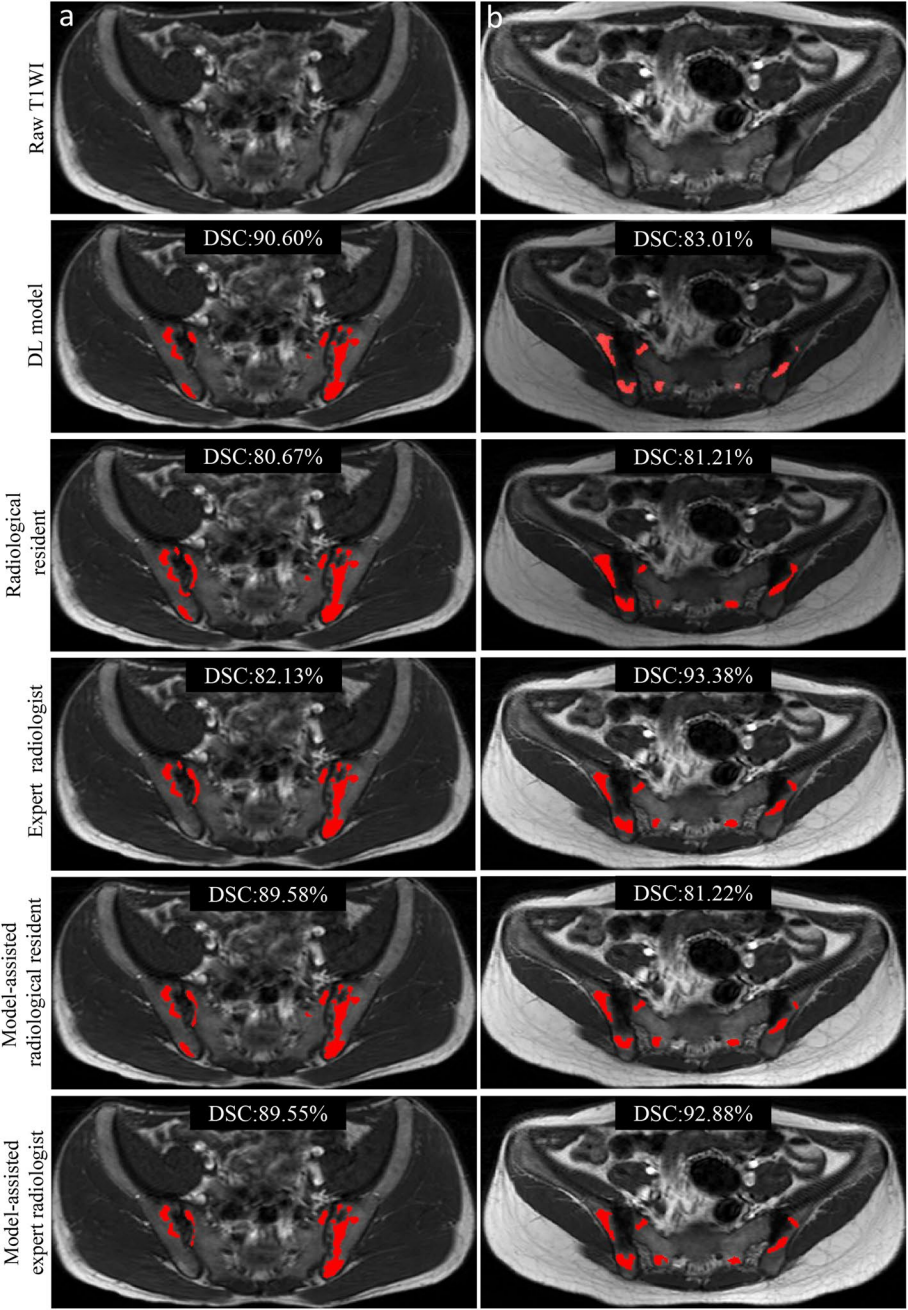
Representative examples of excellent segmentation of fat metaplasia on SIJ MRI by the 2.5D-AttentionUnet segmentation model and radiologists without and with model assistance. **a** A 23-year-old male with axSpA. **b** A 21-year-old female with non-axSpA (osteitis condensans ilii). *axSpA* axial spondyloarthritis; *non-axSpA* non-axial spondyloarthritis; *T1WI* T1-weighted images; *DL* deep learning; *DSC* Dice similarity coefficient

**Fig. 4:**
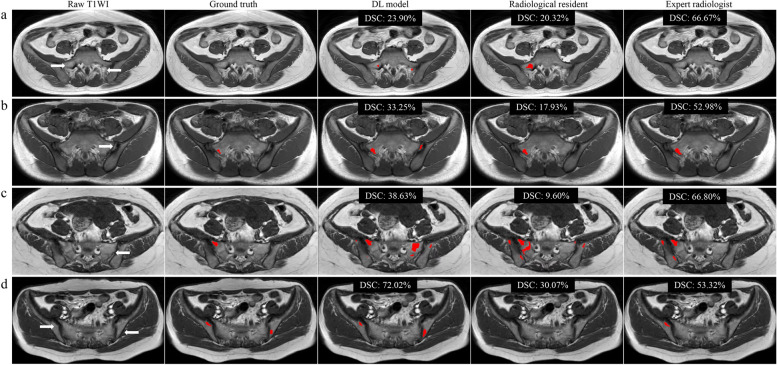
Representative examples of wrong segmentation of fat metaplasia on SIJ MRI by the 2.5D-AttentionUnet segmentation model and radiologists. **a** In a 28-year-old female with axSpA, normal yellow bone marrow fat of the sacrum and normal intermuscular fat were incorrectly segmented by the model and radiological resident (white arrows). **b** In a 17-year-old male with axSpA, the model incorrectly segmented normal fat in the sacroiliac joint space (white arrow) rather than radiologists. **c** In a 27-year-old female with non-axSpA (non-specific sacroiliitis), the model and radiologists incorrectly segmented the normal yellow bone marrow of the sacrum and ilium (white arrows). **d** In a 39-year-old female with axSpA, small patchy FMs in the sacrum and ilium (white arrows) were missed by two radiologists, which are segmented by the model. *axSpA* axial spondyloarthritis; *non-axSpA* non-axial spondyloarthritis; *T1WI* T1-weighted images; *DL* deep learning; *DSC* Dice similarity coefficient

The performance of the 2.5D-AttentionUNet model was superior to that of the radiological resident (DSC, 85.44% vs. 75.70%; *p* < 0.05) and comparable to that of the expert radiologist (DSC, 85.44% vs. 85.03%; *p* > 0.05) on the external test set. With the assistance of the model, the segmentation performance was improved for the radiological resident (DSC, 82.87%, *p* < 0.05; precision, 76.18%) and expert radiologist (DSC, 85.74%, *p* > 0.05; precision, 81.59%) (Table 3). DSCs below 60.00% were obtained in three cases for the radiological resident and a case for the expert radiologist. With the model assistance, radiologists obtained good segmentation performance with DSC higher than 60.00% of all cases. Figures 3 and 4 illustrate representative examples of the excellent and poor segmentation performance of the model and radiologists without and with model assistance.

**Table 3:** Performance of the segmentation deep learning model, radiologists without and with model assistance on the external test set

	DSC (%)	Precision (%)	Recall (%)
2.5D-AttentionUNet model	85.44 ± 6.09	85.83 (82.62–89.04)	86.43 (81.10–91.76)
Radiologists			
Radiological resident	75.70 ± 10.87	66.18 (59.69–72.68)	91.13 (87.71–94.55)
*p* value^a^	0.001	/	/
Expert radiologist	85.03 ± 9.72	80.32 (74.71–85.92)	91.11 (86.84–95.38)
*p* value^a^	0.874	/	/
Model-assisted radiologists			
Radiological resident	82.87 ± 6.88	76.18 (72.01–80.35)	92.05 (88.14–95.95)
*p* value^b^	< 0.001	/	/
Expert radiologist	85.74 ± 8.08	81.59 (76.84–86.33)	91.39 (87.45–95.33)
*p* value^b^	0.496	/	/

DSC is presented as an average percentage with a standard deviation. Precision and recall are shown as percentages with 95% confidential intervals. *p* values less than 0.05 show statistical differences

^a^Data were compared between 2.5D-AttentionUNet model and radiologists

^b^Data were compared between radiologists and model-assisted radiologists. *DSC* Dice similarity coefficient

### Performance of the deep learning classification model

Based on the automatic segmentation results, the classification model achieved AUCs of 0.876 (95% CI: 0.811–0.942) and 0.799 (95% CI: 0.696–0.902) to differentiate axSpA and non-axSpA, with a satisfactory accuracy of 81.25% (95% CI: 73.44–89.06%) and 77.59% (95% CI: 66.85–88.32%) and sensitivity of 88.52% (95% CI: 82.15–94.89%) and 91.89% (95% CI: 84.87–98.92%) on the internal and external test sets, respectively (Table 4). However, the specificity of the model was moderate, with values of 68.57% (95% CI: 59.28–77.86%) and 52.38% (95% CI: 39.53–65.23%). The Grad-CAM heatmap indicated that the most significant area was activated among multiple FM lesions in SIJs, indicating that the classification model correctly recognized the target area. Supplementary Materials I: Appendix Figure 1 illustrates representative examples of the model’s performance in classifying FM on SIJ MRI.

**Table 4:** Performance of the deep learning classification model on the internal and external test sets

Data sets	AUC	Accuracy (%)	Sensitivity (%)	Specificity (%)	PPV (%)	NPV (%)	Cohen’s *κ*
Internal test set	0.876 (0.811–0.942)	81.25 (73.44–89.06)	88.52 (82.15–94.89)	68.57 (59.28–77.86)	83.08 (75.58–90.58)	77.42 (69.06–85.78)	0.504 (0.335–0.673)
External test set	0.799 (0.696–0.902)	77.59 (66.85–88.32)	91.89 (84.87–98.92)	52.38 (39.53–65.23)	77.27 (66.49–88.06)	78.57 (68.01–89.13)	0.477 (0.242–0.712)

All data in parentheses are 95% confidential intervals

*AUC* area under the receiver operating characteristic curve, *PPV* positive predictive value, *NPV* negative predictive value

### Clinical utility of the deep learning classification model

**Fig. 5:**
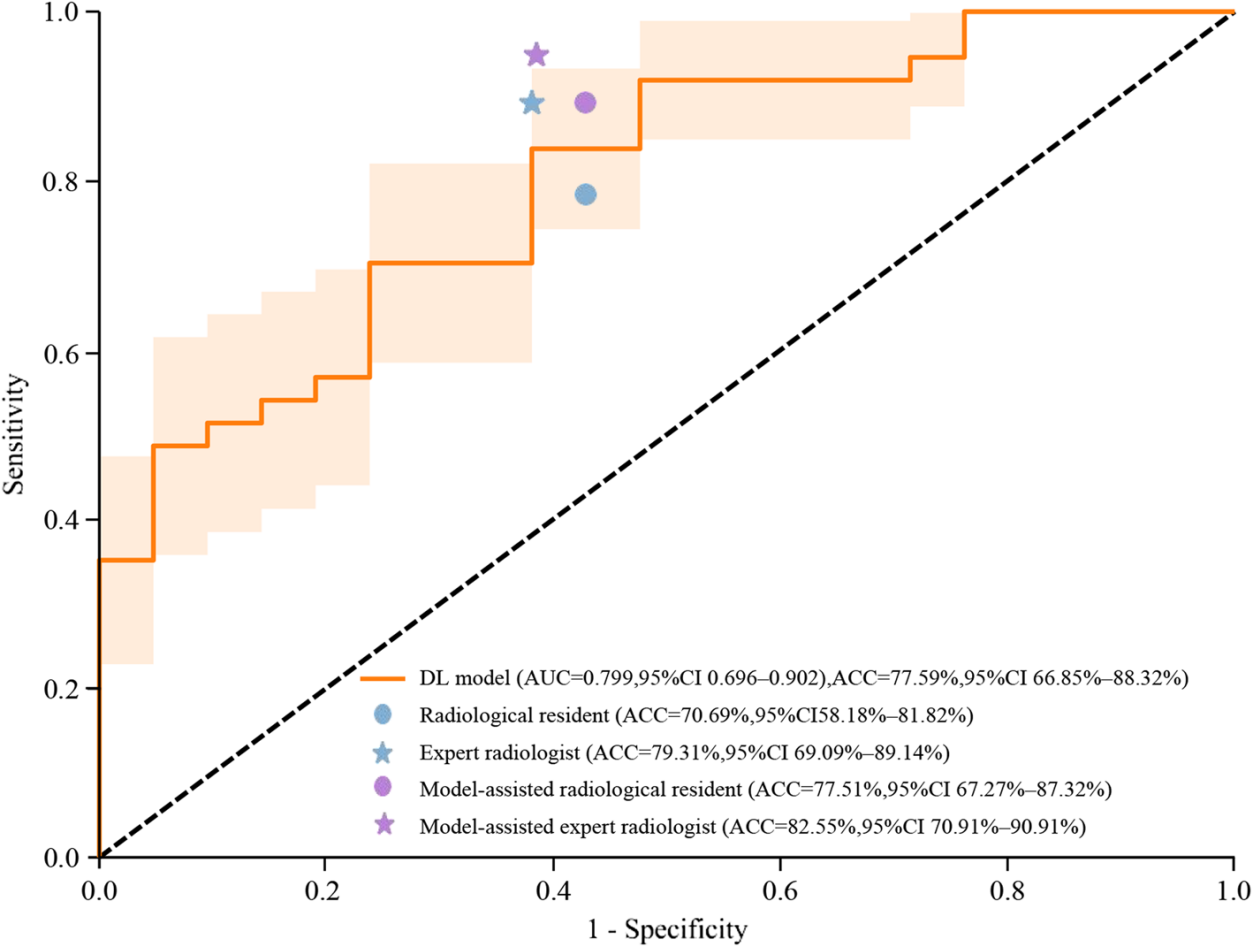
ROC curves of the classification model and performance comparison between radiologists without and with model assistance on the external test set. *DL* deep learning; *ROC* receiver operating characteristic; *AUC* area under the ROC curve; *ACC* accuracy

The accuracy, sensitivity, and specificity of the classification model were superior to those of the radiological resident and the expert radiologist (accuracy, 70.69% and 79.31%; sensitivity, 78.38% and 89.19%; specificity, 57.14% and 61.90%, respectively). With the model assistance, the classification performance was improved for the radiological resident and expert radiologist (accuracy, 77.51% and 82.55%; sensitivity, 89.22% and 94.85%; specificity, 57.20% and 61.51%, respectively; *p* > 0.05) (Table 5 and Fig. 5). Misclassified cases were corrected in five cases for radiological residents and in two cases for expert radiologists with the model assistance.

**Table 5:** Performance of radiologists without and with model assistance on the external test set

Metrics	Radiologists		Model-assisted radiologists			
	Radiological resident	Expert radiologist	Radiological resident	*p* value^a^	Expert radiologist	*P* value^b^
Accuracy (%)	70.69 (58.18–81.82)	79.31 (69.09–89.14)	77.51 (67.27–87.32)	0.572	82.55 (70.91–90.91)	0.152
Sensitivity (%)	78.38 (63.89–91.18)	89.19 (76.92–97.30)	89.22 (78.79–97.44)	0.424	94.85 (85.71–100.00)	0.508
Specificity (%)	57.14 (33.33–78.58)	61.90 (40.72–83.33)	57.20 (34.99–78.57)	0.990	61.51 (40.00–82.36)	0.302
PPV (%)	76.32 (62.16–89.29)	80.49 (68.42–92.50)	78.27 (65.00–90.24)	/	80.87 (68.18–92.50)	/
NPV (%)	60.00 (35.71–82.35)	76.47 (52.94–94.44)	75.34 (52.93–95.00)	/	87.41 (66.67–100.00)	/
Cohen’s *κ*	0.359 (0.082–0.599)	0.533 (0.299–0.766)	0.484 (0.238–0.725)	/	0.596 (0.369–0.802)	/

All data in parentheses are 95% confidential intervals. *p* values less than 0.05 show statistical differences

*AUC* an area under the receiver operating characteristic curve, *PPV* positive predictive value, *NPV* negative predictive value

^a^Data were compared between the radiological resident and model-assisted radiological resident

^b^Data were compared between the expert radiologist and model-assisted expert radiologist

## Discussion

This study developed a novel 2.5D-AttentionUNet-based segmentation to accurately segment FM on SIJ MR images. The models’ performance was validated in two different institutions (internal test: DSC, 81.86%; and external test: DSC, 85.44%) and comparable or even superior to manual segmentation by a radiological resident and an expert radiologist. Based on the segmentation model, a developed DL classification model achieved performance not inferior to that of the two radiologists in distinguishing axSpA from non-axSpA. The effectiveness of the proposed segmentation model was also confirmed as it assisted the two radiologists in achieving better segmentation performance. These results implied that the DL segmentation model could be an effective tool for automatic FM segmentation and be integrated into the automatic analysis workflow for FM on SIJ MRI.

The segmentation of FM is a crucial step in the quantitative analysis of FM (e.g., lesion location, size, and intensity) and helps to understand the association between FM and disease progression [5–7]. However, manual segmentation of FM by radiologists is tedious, time-consuming, and prone to intra- and inter-reader variations [29], which is impractical in daily radiological workflow. Compared to manual segmentation, automatic segmentation has added value that it is very fast and always provide the same results for the same input data. The certainty of automatic tools means that less variability might be introduced than manual methods when monitoring the same patient for disease progression. Our developed automatic tool built a bridge for future quantitative analysis of FM, which is promising to improve patient diagnosis and prognosis and caters to the current trend of personalized medicine.

The histopathology of FM is unknown, however, a recent review reported that macrophage infiltration is common in SIJs of axSpA [30]. The hyperintensity of lesions on SIJ T1WI obviously suggests the presence of lipid. We speculated that the formation of FM in SIJ is due to the involvement of foamy macrophages, a group of cells formed by lipid accumulation and present in chronic inflammation [31, 32]. Compared with developing a segmentation model for normal fat tissue such as abdominal, subcutaneous, and intermuscular fat [33–35], creating a segmentation model for pathological FM in SIJ is more challenging due to its large inter-individual variability, such as irregular size, shape, and distribution. To ensure the validity of the ground-truth segmentation data, we adopted an interaction between the pre-trained model and two expert radiologists to repeatedly calibrate the segmentation masks of FM. This design more closely mimicked clinical routine, in which automated segmentation is first performed by the model and then approved or calibrated by radiologists. Apart from this, it was highly demanded to find a network architecture that was most suitable for FM segmentation task.

We investigated several medical image segmentation networks derived from UNet [24], including 2D-based CNNs (including 2D UNet, ResUNet, Attention UNet), 3D-based CNNs (including 3D UNet and a recent transformer-based UNETR [36]), and our proposed 2.5D-AttentionUNet. These UNet-derived models demonstrated moderate to strong performance for FM segmentation (DSC, 57.81–85.44%) for external testing, indicating that CNN-based automatic segmentation of FM in SIJs is feasible. However, the performance of 2.5D-AttentionUNet was superior to that of 2D- and 3D-based CNNs. This may be due to two reasons. First, 2D-based CNNs effectively extracted in-plane features of FM in each MR slice, but ignored the spatial distribution information of FM in different MR slices. Second, using 3D-based CNNs directly was limited because the different in-plane and through-plane resolutions of MR images yield varying information densities. Our 2.5D-AttentionUNet-based model fully leveraged in-plane and within-slice features of MR slices, and was therefore well-suited for the FM segmentation task to provide accurate lesion segmentation.

Previous studies developed DL models to detect different types of inflammation or structural damage of axSpA, with AUCs of 0.76–0.98 for bone marrow edema on SIJ MRI [37, 38], 0.92 for erosion, and 0.91 for ankylosis on SIJ CT [15]. Our experimental results demonstrated the effective application of DL methods in FM, filling a critical gap in the automatic identification of SIJ changes reflecting axSpA progression (i.e., inflammation-erosion-FM-new bone formation) [39]. High value of DL models has been confirmed in identifying inflammation and structural damage indicative of axSpA based on SIJ image analysis [38, 40]. However, the previous researchers did not independently analyze FM. In addition, none of the previous studies has evaluated the clinical utility of DL models as an auxiliary tool to assist radiologists in interpreting SIJ changes on MRI.

We asked radiologists to interact with the automatic tool to simulate the process of shared decision-making and initially explore the application of automatic FM segmentation in assisting axSpA diagnosing. The model assistance added value to the identification of FM by reducing radiologists’ interpretation errors. For example, scattered and small FM lesions with poorly defined margins on SIJ MRI were incorrectly segmented by radiologists but accurately segmented by the DL model. These FM lesions were challenging to be identified by the naked eye [10, 11], but their representative features could be learned by the DL model through iterative optimization (Figs. 3 and 4d). However, radiologists and our classification model all exhibited low specificity (52.38–61.90%) because they tend to misclassify non-axSpA as axSpA. This may be attributed to the lack of clinical indicators specific to disease diagnosis in the classification task, such as HLA-B27 status, age, and gender.

Unfortunately, incorrect results are still produced in some cases. A small part of normal fat in the sacral foramen, SIJ space, and intermuscular space was misidentified as FM. This fat can be identified by the expert radiologist, but was wrongly identified by the radiological resident (Fig. 4a and b). In addition, another segmentation error occurred when FM coexisted with multiple types of other SIJ changes (Fig. 4c). On T1WI, bone marrow edema decreases the intensity of FM, and erosion and sclerosis changed the shape of FM [1, 3]. On the contrary, experienced radiologists accurately segmented FM through observing overall SIJ MRI changes. These findings suggested that the DL model can assist physicians in interpreting FM, but cannot directly replace them in routine clinical settings.

Our study had some limitations. First, this retrospective study may lead to patient selection bias. Second, our segmentation and classification models were developed using axial T1WI only, which might limit the model’s generalization in different hospitals. In future studies, we will use images obtained from multiple MRI sequences and orientations to develop more robust models. Third, the reference standard for FM on SIJ MRI was determined by consensus among four radiologists without pathological confirmation, because pathological biopsy is usually unnecessary in such patients. It might introduce noise into annotations of FM and compromise the model’s performance. Fourth, we did not include healthy populations or patients with non-rheumatic diseases (such as herniated discs and infection) for model development, which might reduce the applicability of our models in real-world practice. In the future, we will enroll patients with various diseases and healthy populations to optimize DL models to meet clinical needs better.

## Conclusion

In conclusion, the novel 2.5D-AttentionUnet-based model automatically and effectively segmented FM on SIJ MRI. The model is promising to serve as an auxiliary tool for radiologists to quantitatively analyze FM on SIJ MRI, which has the potential to improve diagnosis and prognosis for axSpA in routine clinical settings.

### Supplementary Information

**Supplementary Material 1.**

## Data Availability

The datasets used and/or analyzed during the current study are available from the corresponding author on reasonable request.

## Abbreviations

ASAS: Assessment in Spondyloarthritis International Society
AUC: Area under the receiver operating characteristic curve
axSpA: Axial spondyloarthritis
CI: Confidence intervals
CNN: Convolutional neural networks
CRP: C-reactive protein
DICOM: Digital imaging and communications in medicine
DL: Deep learning
DSC: Dice similarity coefficient
ESR: Erythrocyte sedimentation rate
FM: Fat metaplasia
FS: Fat-saturated
HLA-B27: Human leukocyte antigen-B27
ICC: Intraclass correlation coefficients
MRI: Magnetic resonance image
NPV: Negative predictive value
PPV: Positive predictive value
ROI: Region of interest
SIJs: Sacroiliac joints
T1WI: T1-weighted images
T2WI: T2-weighted images
