# Supplementary material for: Automatic segmentation of fat metaplasia on sacroiliac joint MRI using deep learning
Source: Insights Imaging. 2024 Mar 26;15:93. doi: 10.1186/s13244-024-01659-y (PMC10965870; doi:10.1186/s13244-024-01659-y)
Supplement: Supplementary file 1 — Supplementary Material 1. [file 13244_2024_1659_MOESM1_ESM.docx]

**Automatic segmentation of** **fat metaplasia indicative of axial spondyloarthritis on** **sacroiliac joint MRI using deep learning**

**Appendix material**

**Appendix Table 1 Detailed disease subtypes of patients with non-axial spondyloarthropathy**

**Appendix Table 2 MRI protocol**

**Appendix Table 3 Patient characteristics for the classification task**

**Appendix Figure 1 Examples of Grad-CAM for the deep learning model**

**Appendix method 1. The evaluation criteria of fat metaplasia in sacroiliac joints on MRI**

**Appendix method 2. Preprocessing**

**Appendix method 3. The novel 2.5D-AttentionUNet segmentation model development**

**Appendix method 4. Deep learning classification model development**

**Appendix Table 1** Detailed disease subtypes of patients with non-axial spondyloarthropathy

| Disease subtype | Center 1 (n = 186) | Center 2 (n = 21) |
| --- | --- | --- |
| Non-specific sacroiliitis | 59 (31.7) | 14 (66.7) |
| Rheumatoid arthritis | 4 (2.2) | 1 (4.8) |
| Osteitis condensans ilii | 3 (1.6) | 2 (9.5) |
| Gouty arthritis | 6 (3.2) | 3 (14.3) |
| [Psoriatic arthritis](javascript:;) | 6 (3.2) | 1 (4.7) |
| Degenerative arthritis | 2 (1.1) | 0 (0.0) |
| Peripheral spondyloarthritis | 68 (36.6) | 0 (0.0) |
| Systemic lupus erythematosus | 1 (0.5) | 0 (0.0) |
| Undifferentiated spondyloarthritis | 37 (19.9) | 0 (0.0) |
| Data in parentheses are percentages. *n* Number. | | |

**Appendix Table 2** MRI protocol

| Parameters | Centre 1 | | Centre 2 |
| --- | --- | --- | --- |
| Field strength | Philips 1.5T (Achieva) | Philips 3.0T (Ingenia) | Philips 3.0T (Achieva) |
| Sequence | Axial T1WI | Axial T1WI | Axial T1WI |
| TE/TR | 18/503 | 20/664 | 8/529 |
| Slice (mm) | 6.0 | 5.0 | 7.0 |
| Gap (mm) | 7.0 | 5.5 | 7.0 |
| FOV (mm) | 196 | 175 | 198 |
| Matrix (mm) | 356 × 280 | 476 × 351 | 417 × 345 |

*TE* echo time, *TR* repetition time, *T1WI* T1-weighted imaging, *mm* millimeter, *FOV* field of view.

**Appendix Table 3** Patient characteristics for the classification task

| Characteristics | All  (n = 706) | Training set  (n = 455) | Validation set  (n = 64) | Internal test set  (n = 129) | External test set  (n = 58) | *p* value |
| --- | --- | --- | --- | --- | --- | --- |
| Age (years) | 28.0  (23.0, 34.0) | 28.0  (24.0, 34.0) | 26.5  (22.0, 30.8) | 29.0  (24.0, 36.0) | 24.0  (20.0, 4.0) | 0.378 |
| Disease duration (months) | 24.0  (6.0, 72.0) | 24.0  (8.0, 72.0) | 36.0  (9.0, 66.0) | 36.0  (9.5, 87.0) | 11.0  (3.0, 72.0) | 0.134 |
| Sex |  |  |  |  |  | 0.775 |
| male | 519 (73.5) | 335 (73.6) | 46 (71.9) | 98 (76.0) | 40 (68.9) |  |
| female | 187 (26.5) | 120 (26.4) | 18 (28.1) | 31 (24.0) | 18 (31.1) |  |
| HLA-B27 |  |  |  |  |  | 0.045 |
| (+) | 360 (51.0) | 235 (51.6) | 39 (60.9) | 58 (45.0) | 28 (48.3) |  |
| (-) | 179 (25.4) | 116 (25.5) | 17 (26.6) | 41 (31, 8) | 5 (8.6) |  |
| Missing | 167 (23.6) | 104 (22.9) | 8 (12.5) | 30 (23.2) | 25 (43.1) |  |
| ESR (mm/H) | 17.0 (7.0, 36.8) | 16.0 (7.0, 34.0) | 26.5 (15.0, 38.0) | 13.0 (6.0, 31.0) | 49.0 (27.0, 64.0) | < 0.001 |
| CRP (mg/L) | 7.0 (2.00, 19.0) | 6.0 (1.61, 18.00) | 9.5 (5.00, 21.0) | 6.0 (1.07, 13.9) | 25.7 (9.2, 72.0) | < 0.001 |

Categorical variables are presented as number with percentage in parentheses, and continuous variables are shown as median with interquartile range in parentheses. *HLA-B27* human leukocyte antigen-B27, *ESR* erythrocyte sedimentation rate, *CRP* C-reactive protein, *mm/H* millimeter per hour, *mg/L* milligram per liter, *n* number.

**Appendix Figure 1.** Examples of Grad-CAM for the DL model


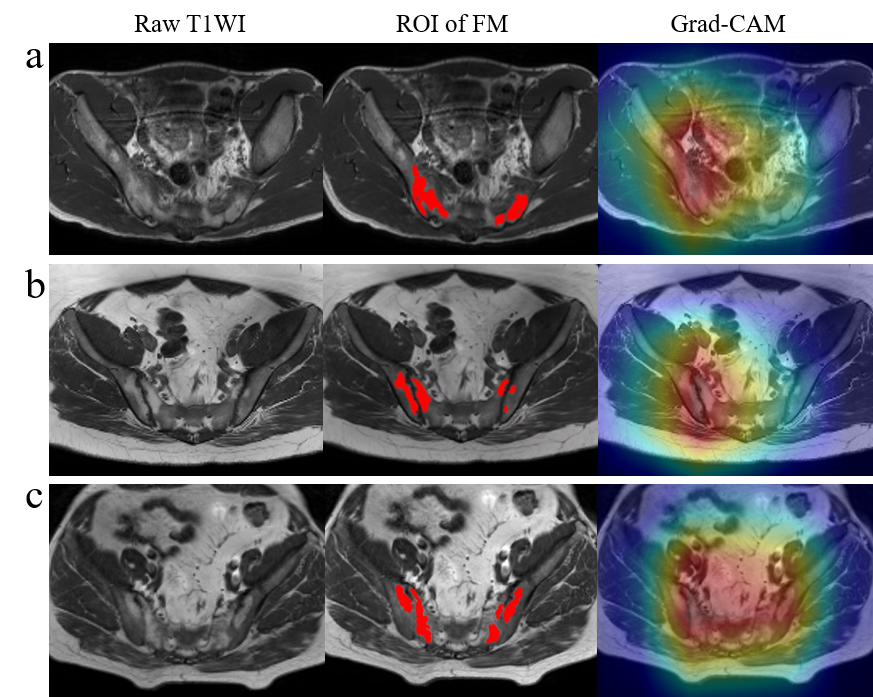


(a) a 33-year-old male with axSpA. (b) a 30-year-old male with gouty arthritis (non-axSpA). (c) a 37-year-old male with axSpA. The highlight visuals reflect the region of interest of FM limited to unilateral (right) sacroiliac joints when predicted at the time of prediction (a, b). The DL model is overfitting, and the highlighted vision is located in the center of the pelvis, covering the bilateral sacroiliac joints (c). *T1WI,* T1-weighted image; *ROI,* region of interest; *DL,* deep learning; *Grad-CAM,* Gradient-weighted Class Activation Mapping; *axSpA,* axial spondyloarthritis; *non-axSpA*, non-axial spondyloarthritis; *FM,* fat metaplasia

**Appendix method 1. The evaluation criteria of** **fat metaplasia in sacroiliac joints on MRI**

The Assessment of SpondyloArthritis International Society (ASAS) MRI working group defined fat metaplasia (FM) in sacroiliac joints (SIJs) of patients with axial spondyloarthritis (axSpA) as a large signal area with a sharp border in the subchondral bone that is homogeneously brighter than normal bone marrow on axial T1-weighted image (T1WI) and T2-weighted image (T2WI), and hypointensity on fat-saturated (FS) T2WI, noteworthily, a few lesions with blurred edges [1]. For non-axSpA patients, FM was defined as scattered patchy lesions with blurred or sharp edges in the subchondral bone on SIJ MRI with the same signal as axSpA patients.

**Appendix method 2. Preprocessing**

Preprocessing was performed using the SimpleITK library (version 2.1.1). Each MRI was interpolated into a voxel spacing of 1.0 mm × 1.0 mm × 7.0 mm. Intensity normalization was applied to each scan, as the scans were large in volume and variant in shape. To address the class imbalance problem, we randomly cropped three sub-volumes of 192 × 192 × 16 voxels at a positive: negative ratio 1:1 for each scan. We added data augmentation strategies to tolerate variances in the location, size, and orientation of FM in different patients, including random rotation, random scale, random flip, and random Gaussian noise. Intensity values were normalized to zero-mean and unit-variance intensities for individual volumes.

**Appendix method 3. The novel 2.5D-AttentionUNet segmentation model development**

Our novel segmentation model first used two-dimensional (2D) convolution and 2D down-sampling until the resolution of all axes was approximately the same. We then used three-dimensional (3D) convolution to capture the whole-volume feature, which leveraged the advantages of both 2D and 3D convolution.

Our model mainly followed the structure of U-Net [2]. The encoder contained five levels of convolution. The first three levels use 2D convolution/max pooling. The other two levels used 3D convolution/max pooling based on the fact that the in-plane resolution of our data after resampling was seven times greater than the through-plane resolution (i.e., 1 mm, 1 mm and 7 mm for the x-, y- and z-axis, respectively). At each level, the block design was the same as that used for an efficient context-aware network [3], which features residual connection. At the bottleneck encoder, we used the mixed pooling module described by Hou et al. [4] to encode the global context, motivated by the variation in the shape of the fat deposits in the data. We used the same decoder design as an efficient context-aware network [3], which features anisotropic kernels to save memory and slim architecture by leveraging the power of encoders. Spatial attention [5] captured low-level features, such as boundaries, while squeeze-and-attention [6] distinguished the relative importance of the feature maps obtained at deeper levels. Our loss functions for training were:

$L_{\mathrm{CE}}=-\frac{1}{N}\sum_{i=0}^{N} p_{i}*\log(q_{i})$, (1)

where $p_{i}$and $q_{i}$ denote the ground truth and the prediction value for voxel $i$, respectively;

$L_{\mathrm{Dice}}= 1-\frac{\left( 2\sum_{i=1}^{I} G_{i} *Y_{i} \right)}{\sum_{i=1}^{I} G_{i}+\sum_{i=1}^{I} Y_{i}}$ , (2)

where $G_{i}$ and $Y_{i}$ denote the ground truth and the prediction value for voxel $i$, respectively;

$L^{'}=L_{\mathrm{Dice}}+L_{\mathrm{CE}}$, (3)

$L_{\mathrm{Attention}}=L^{'}\left( A_{m},G_{m} \right)$, (4)

where $A_{m}$ is the attention map at level $m$ and $G_{m}$ is the average pooling of the ground truth, which has the same resolution as $A_{m}$; and

$L=L_{\mathrm{Attention}}+L_{\mathrm{Dice}}+L_{\mathrm{CE}}$, (5)

where $L$ is the total loss.

**Appendix method 4. Deep learning classification model development**

With a few minor exceptions, the segmentation process of the classification model was almost identical to the segmentation process of the segmentation model. The target area could be isolated from the network because it is relatively fixed where fat deposits are located. Therefore, a region-of-interest crop size of [53, 53, 1] to [303, 303, 27] was implemented following a non-randomized sequence. This technique enabled the network to disregard background data and concentrate on the fat distribution, which is more vulnerable to variation due to slight textural variations between axSpA and non-axSpA. Furthermore, the random crop by the centers’ positive: negative ratio was eliminated because the positive and negative samples were no longer voxels, but were images.

We added a 1000-2 multilayer perceptron (MLP) to the end of the previous 2.5D-UNet baseline network for classification purposes, and binary cross-entropy loss was used. In the MLP structure, which consisted of two sequential, fully connected layers, the first 1,000 neurons took results from the UNet that had been flattened, while the following two neurons acted as the classifier. The softmax function turned the output of the final two neurons into probabilities, given by:

$$\sigma\left( \vec{z} \right)_{i}=\frac{e^{z_{i}}}{\sum_{j=1}^{K} e^{z_{j}}}$$

where $\vec{z}$ is the output of the two neurons, and $K$ is the number of classes.

The loss function was the cross-entropy’s binary version as follows:

$$loss=\sum_{j=1}^{K} \log\sigma\left( \vec{z} \right)_{1}$$

**Reference**

1. Maksymowych WP, Lambert RG, Ostergaard M et al (2019) MRI lesions in the sacroiliac joints of patients with spondyloarthritis: an update of definitions and validation by the ASAS MRI working group. Ann Rheum Dis 78:1550-1558. <https://doi.org/10.1136/annrheumdis-2019-215589>

2. Falk T, Mai D, Bensch R et al (2019) U-Net: deep learning for cell counting, detection, and morphometry. Nat Methods 16:67-70. <https://doi.org/10.1038/s41592-018-0261-2>

3. Zhang F, Wang Y, Yang H (2021) Efficient context-aware network for abdominal multi-organ segmentation. arXiv preprint arXiv:210910601

4. Hou Q, Zhang L, Cheng M-M, Feng J (2020) Strip pooling: Rethinking spatial pooling for scene parsingProceedings of the IEEE/CVF Conference on Computer Vision and Pattern Recognition, pp 4003-4012

5. Zhao T, Wu X (2019) Pyramid feature attention network for saliency detectionProceedings of the IEEE/CVF conference on computer vision and pattern recognition, pp 3085-3094

6. Zhong Z, Lin ZQ, Bidart R et al (2020) Squeeze-and-attention networks for semantic segmentationProceedings of the IEEE/CVF conference on computer vision and pattern recognition, pp 13065-13074
